# A systematic review and meta-analysis of randomized controlled trials on the effects of neuromuscular electrical stimulation in patients with acute heart failure

**DOI:** 10.7717/peerj.21352

**Published:** 2026-06-09

**Authors:** Fu-An Yang, Yu-Han Hong, Ya-Ling Tseng, Dorji Harnod, Hung-Chou Chen

**Affiliations:** 1 Department of Physical Medicine and Rehabilitation, Far Eastern Memorial Hospital, New Taipei City, Taiwan; 2 School of Medicine, College of Medicine, Taipei Medical University, Taipei, Taiwan; 3 Department of Physical Medicine and Rehabilitation, Shuang Ho Hospital, Taipei Medical University, New Taipei City, Taiwan; 4 Department of Emergency, School of Medicine, College of Medicine, Taipei Medical University, Taipei, Taiwan; 5 Division of Critical Care Medicine, Department of Emergency and Critical Care Medicine, Shuang Ho Hospital, Taipei Medical University, New Taipei City, Taiwan; 6 Department of Physical Medicine and Rehabilitation, School of Medicine, College of Medicine, Taipei Medical University, Taipei, Taiwan; 7 Center for Evidence-Based Health Care, Shuang Ho Hospital, Taipei Medical University, New Taipei City, Taiwan

**Keywords:** Acute heart failure, Electrical stimulation, Meta-analysis, Neuromuscular electrical stimulation, Systematic review

## Abstract

**Background:**

Acute heart failure often leads to impaired physical function, high rehospitalization rates, and poor quality of life. Although exercise-based rehabilitation benefits chronic heart failure patients, its feasibility in acute heart failure is limited. Neuromuscular electrical stimulation offers a potential alternative by safely inducing muscle contractions without causing dyspnea.

**Methods:**

The protocol was registered with the International Prospective Register of Systematic Reviews (registration number CRD42023453116). Following PRISMA guidelines, a comprehensive search of PubMed, Cochrane Library, and Embase was conducted up to October 13, 2025. Randomized controlled trials comparing neuromuscular electrical stimulation to control treatments in patients with acute heart failure were included. Data synthesis was performed using Review Manager 5.4.

**Results:**

Seven randomized controlled trials, with methodological quality ranging from fair to excellent (Physiotherapy Evidence Database (PEDro) scores 5–9), were included. Pooled data analysis revealed that neuromuscular electrical stimulation significantly improved 6-min walking distance (mean difference = 69.92 m, 95% confidence interval CI [32.17–1 07.68], * p* = 0.0003), quality of life (standardized mean difference = 1.53, 95% CI [1.03–2.03], *p* < 0.00001), and showed preliminary evidence of improvement in leg muscle strength (standardized mean difference = 0.77, 95% CI [0.25–1.29], *p* = 0.004), whereas no significant difference was observed in left ventricular ejection fraction (mean difference = 1.94%, 95% CI [−3.91 to 7.79], * p* = 0.52). Neuromuscular electrical stimulation was generally well tolerated, with no serious adverse events directly attributable to the intervention.

**Conclusion:**

Neuromuscular electrical stimulation was noted to be effective for improving physical capacity and quality of life in patients with acute heart failure. It offers a promising option for patients unable to engage in conventional rehabilitation. Further large-scale, multicenter Randomized Controlled Trials (RCTs) are needed to confirm these findings.

## Introduction

Acute heart failure is defined as rapid changes in symptoms and signs of heart failure that require urgent medical attention (Arrigo et al., 2020). It is often associated with poor health-related quality of life, frequent rehospitalization, increased financial burden, high mortality, and immobilization due to hemodynamic instability and severe dyspnea, which further contributes to muscle wasting and atrophy (Kitzman et al., 2021; Pandey et al., 2019). Acute heart failure is the leading cause of hospitalization among older people, with approximately one million admissions per year in the United States and Europe (Mozaffarian et al., 2015). As a result, severe physical function impairment is commonly observed in this population (Warraich et al., 2018).

The management and prevention of the sequelae of acute heart failure are crucial. Recent meta-analyses have indicated that exercise-based cardiac rehabilitation improves quality of life and exercise capacity in patients with chronic heart failure (Kitzman et al., 2021; Long et al., 2019). In addition, greater improvements in physical function, a higher likelihood of discharge, and shorter hospital stays have been reported among individuals who undergo early progressive rehabilitation involving multiple domains of physical function compared with those receiving usual care (Kamiya et al., 2020; Chun & Kang, 2021; Fleming et al., 2018). Current guidelines recommend comprehensive cardiac rehabilitation for patients with stable heart failure (Ponikowski et al., 2016; Bozkurt et al., 2021). However, exercise training capabilities are limited in many patients with heart failure because of the markedly reduced cardiac and pulmonary reserves and peripheral muscle dysfunction (Del Buono et al., 2019).

Neuromuscular electrical stimulation can safely induce muscle contractions without eliciting dyspnea even in patients with critical illness and heart failure (Ennis et al., 2017; Iwatsu et al., 2015; Segers et al., 2014; Kondo et al., 2019). It also improves cardiopulmonary function and health-related quality of life in patients with chronic heart failure (Wang et al., 2022; Gomes Neto et al., 2016; Sbruzzi et al., 2010). Although it benefits outpatients with stable moderate heart failure, its short-term efficacy in advanced stages of heart failure or in acute care settings remains inconclusive (Wang et al., 2022; Neto et al., 2016; Sbruzzi et al., 2010). While several meta-analyses have confirmed the benefits of neuromuscular electrical stimulation in patients with stable chronic heart failure, this population is clinically distinct from those in an acute decompensated state. Patients with acute heart failure often suffer from severe immobilization, hemodynamic instability, and profound muscle wasting, which limits the feasibility of conventional rehabilitation. To date, the evidence for neuromuscular electrical stimulation as a safe and effective intervention during this critical acute hospitalization phase has not been systematically synthesized. Therefore, this systematic review and meta-analysis was conducted to evaluate the effects of neuromuscular electrical stimulation on functional capacity, quality of life, and muscle strength specifically in patients hospitalized with acute heart failure.

## Methods

This systematic review was conducted in accordance with the methodological guidance outlined in the Cochrane Handbook for Systematic Reviews of Interventions (Higgins et al., 2024). The reporting of this research followed the Preferred Reporting Items for Systematic Reviews and Meta-Analysis (PRISMA) guidelines (Page et al., 2021). PRISMA checklist was provided (Page et al., 2021). The protocol was registered with the International Prospective Register of Systematic Reviews (registration number CRD42023453116). The initial screening began before PROSPERO registration because we conducted pilot work and a feasibility check during protocol preparation. At that stage, only preliminary title/abstract screening was performed to verify the applicability of the eligibility criteria. No full-text screening, data extraction, risk-of-bias assessment, or data synthesis was conducted prior to the PROSPERO registration date. All formal review procedures followed the registered protocol.

The studies were selected on the basis of the population–intervention–comparison–outcome–study design (PICOS) strategy.

• Population: hospitalized patients (men or women) with acute heart failure
• Intervention and comparison: the efficacy of neuromuscular electrical stimulation compared with control treatments (early conventional rehabilitation, usual care, or sham neuromuscular electrical stimulation) applied to the legs of hospitalized patients
• Outcomes: functional capacity (assessed using 6-min walking distance (6MWD)), quality of life (assessed using various questionnaires), leg muscle strength, and left ventricular ejection fraction (LVEF)
• Study design: only RCTs, no language restrictions

The PubMed, Cochrane Library, and Excerpta Medica (Embase) databases were searched from inception until October 13, 2025, for relevant RCTs (The earliest publication years of articles included in each database were 1946 for PubMed, 1947 for Embase, and 1948 for the Cochrane Library). The keywords were divided into two parts. The first part included “heart failure,” “HF,” “CHF,” and “AHF” for patient type, and the second part comprised “electrical stimulation,” “electric* AND stimulation,” “electrostimulation,” “FES,” “NMES,” and “EMS” for the intervention category. The corresponding MeSH terms of “heart failure” and “electrical stimulation” were searched in the PubMed database and later adapted for the other databases. RCTs were identified by applying the “Randomized Controlled Trial” filter in PubMed and Embase databases. For the Cochrane Library, Randomized Controlled Trials (RCTs) were identified with additional search term, “random*.”

Systematic reviews of similar topics were also reviewed to identify additional studies. All databases were searched from the time of their inception to October 13, 2025. First, two independent researchers (Fu-An Yang and Yu-Han Hong) screened the selected studies based on their titles and abstracts to identify potentially relevant RCTs, followed by full-text reviews. Any discrepancies were resolved through consultation with the third researcher (Hung-Chou Chen).

Studies were included if they (1) were RCTs (2) involved hospitalized patients (3) involved patients diagnosed with acute heart failure and (4) involved treatment with neuromuscular electrical stimulation as the intervention and (5) reported outcomes, including functional capacity (6MWD), leg muscle strength, quality of life, and hemodynamic parameters. Articles were excluded if they were protocols, non-peer-reviewed articles, conference papers, and letters to the editor. No language restriction was applied. Any disagreements were resolved through discussion with a third researcher.

The following data were extracted from the eligible studies by the researchers: sample size, age, sex, New York Heart Association functional classification, LVEF, information regarding the intervention plan (stimulus position, frequency, and duration), the parameters of the stimulator (intensity and pulse width), and outcome measures. In addition, data concerning adverse events or side effects reported in each included study were extracted. Outcomes were included if they provided a point estimate and a measure of variability. Missing data were requested from the study authors by email.

Two assessors independently examined the methodological quality of the studies by employing the 11-item Physiotherapy Evidence Database (PEDro) scale, a valid and reliable tool for assessing the quality of physiotherapy trials (Cashin & McAuley, 2020). The total score ranges from 0–10 and was graded as follows: poor (0–3), fair (4–5), good (6–8), and excellent (9–10) (Cashin & McAuley, 2020). Any disagreements were resolved through discussion with a third reviewer.

The primary outcome was the 6MWD, which is used to assess an individual’s functional status and is used for prognostic evaluation in patients with heart failure (Balady et al., 2010). The secondary outcomes were quality of life, leg muscle strength, and LVEF. Only outcomes presented in more than two RCTs were pooled into quantitative analysis.

The meta-analysis was performed with RevMan (version 5.4; The Cochrane Collaboration, London, United Kingdom). The study was performed according to PRISMA guidelines (Page et al., 2021). All data from each selected RCT were pooled in the statistical analysis, and the results are presented as mean differences (MDs). Relevant data measured using different scales were converted to a single scale by using standard mean difference (SMD). The precision of effect sizes was reported as 95% confidence intervals (CIs). Given the clinical and methodological diversity among the studies, a random-effects model meta-analysis was applied to analyze the pooled data for all evaluated outcomes. Additionally, the degree of statistical heterogeneity among the studies, which is regarded as high at >75%, was assessed using the *I*^2^ test. Statistical significance was *p* <  0.05.

The overall certainty of evidence for each outcome was evaluated using the Grading of Recommendations Assessment, Development and Evaluation (GRADE) framework (Guyatt et al., 2008). The evidence was downgraded based on five domains: risk of bias, inconsistency, indirectness, imprecision, and publication bias. The certainty of evidence was categorized as high, moderate, low, or very low.

## Results

**Figure 1:**
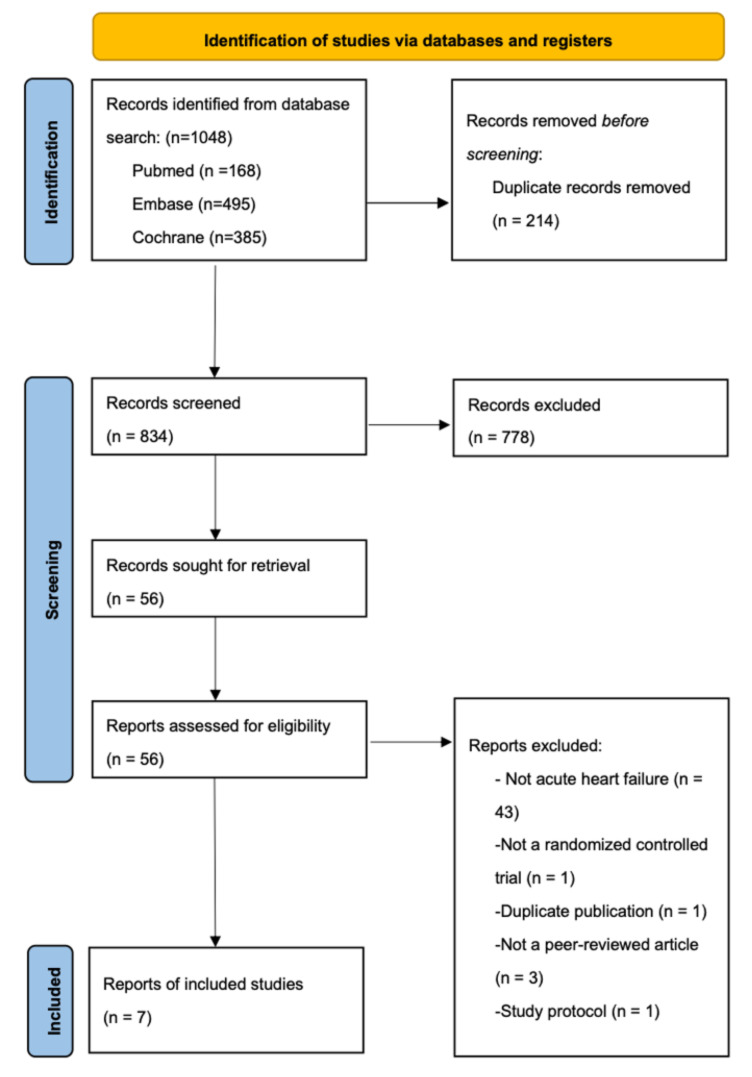
PRISMA flow diagram of trial selection.

The initial database search, conducted on October 13, 2025, across PubMed (*n* = 168), EMBASE (*n* = 495), and the Cochrane Library (*n* = 385) yielded a total of 1048 records. After the removal of 214 duplicates using EndNote (EndNote V, 2013), the titles and abstracts of the remaining 834 unique records were screened for relevance. During this initial screening, 778 records were excluded. The full texts of the remaining 56 articles were then assessed in detail against the eligibility criteria. This second screening stage resulted in the exclusion of a further 49 articles. Ultimately, a total of seven studies met the eligibility criteria and were included in both the systematic review and the quantitative meta-analysis (Kondo et al., 2019; Arenja et al., 2021; De Araújo et al., 2012; Forestieri et al., 2018; Groehs et al., 2016; Poltavskaya et al., 2022; Tanaka et al., 2022). All of them compared the effects of neuromuscular electrical stimulation of the legs with control treatments among patients with acute heart failure. Figure 1 presents the PRISMA flow diagram of study selection and lists the reasons for exclusion (Page et al., 2021). A total of 124 and 132 patients were included in the intervention and control groups, respectively, with a noticeable male predominance across most of the included trials. The patient characteristics and protocols of neuromuscular electrical stimulation from each selected trial are summarized in Tables 1 and 2, respectively. Arenja et al. (2021) randomized the participants into three groups: low-intensity training, high-intensity training, and control groups. The low-intensity group was used as the intervention group because of the protocol’s similarity to those in other studies and compared it with the control group.

The PEDro scale scores for the seven RCTs ranged from 5 to 9, indicating that the methodological quality was fair to excellent. Specifically, the quality of two studies was rated as ‘fair’ (Arenja et al., 2021; Groehs et al., 2016), four were rated as ‘good’ (Kondo et al., 2019; De Araújo et al., 2012; Forestieri et al., 2018; Tanaka et al., 2022), and one was ‘excellent’ (Poltavskaya et al., 2022). Table 3 presents the detailed methodological quality assessment for each included study.

As for the outcome measurement, 6MWD was evaluated to represent patients’ cardiopulmonary function which also served as the primary outcome of this study. The patients’ quality of life was assessed using the Minnesota Living with Heart Failure Questionnaire (MLHFQ) and EuroQol-visual analog scales (EQ-VAS); leg muscle strength (*e.g.*, quadriceps isometric strength (QIS)) and LVEF were measured using a dynamometer and an echocardiogram, respectively.

Because the units of reported data were not uniform across the studies, SMD was used to measure the quality of life and leg muscle strength. The units of reported data for the 6MWD and LVEF were consistent across studies; thus, MD was used to compare the control and intervention groups.

**Figure 2:**
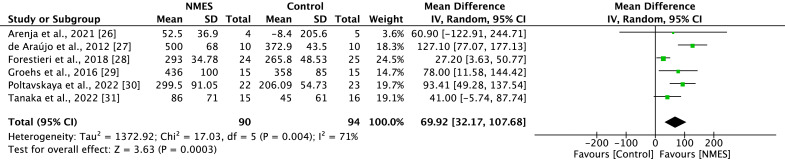
Comparing the effects of neuromuscular electrical stimulation with control groups on 6-min walking distance. Abbreviations: CI, confidence interval; NMES, neuromuscular electrical stimulation.

**Figure 3:**
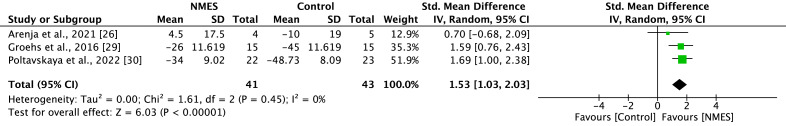
Comparing the effects of neuromuscular electrical stimulation with control groups on patients’ quality of life. Abbreviations: CI, confidence interval; NMES, neuromuscular electrical stimulation; SMD, standardized mean difference.

**Figure 4:**
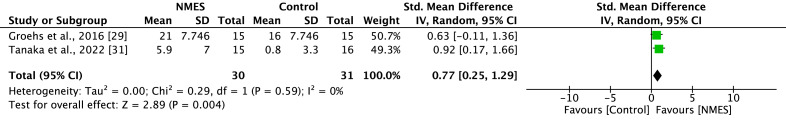
Comparing the effects of neuromuscular electrical stimulation with control groups on leg muscle strength. Abbreviations: CI, confidence interval; NMES, neuromuscular electrical stimulation; SMD, standardized mean difference.

**Figure 5:**
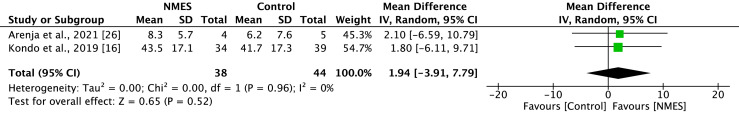
Comparing the effects of neuromuscular electrical stimulation with control groups on left ventricular ejection fraction. Abbreviations: CI, confidence interval; NMES, neuromuscular electrical stimulation.

The primary outcome was reported in six studies (Arenja et al., 2021; De Araújo et al., 2012; Forestieri et al., 2018; Groehs et al., 2016; Poltavskaya et al., 2022; Tanaka et al., 2022), with 90 and 94 patients in the intervention and control groups, respectively. Figure 2 indicated that the intervention groups had a significantly increased functional capacity compared with the control groups (MD = 69.92 m, 95% CI [32.17–107.68], *p* = 0.0003, and *I*^2^ = 71%). The patients’ quality of life was assessed in three studies, (Arenja et al., 2021; Groehs et al., 2016; Poltavskaya et al., 2022) with 41 and 43 participants in the intervention and control groups, respectively (Fig. 3). The intervention group had significantly better quality of life than did the control group (SMD = 1.53, 95% CI [1.03–2.03], *p* < 0.00001, and *I*^2^ = 0%). Two studies presented the results of leg muscle strength (Groehs et al., 2016; Tanaka et al., 2022), with 30 and 31 patients in the intervention and control groups, respectively (Fig. 4). The intervention group had significantly higher leg muscle strength than did the control group (SMD = 0.77, 95% CI [0.25–1.29], *p* = 0.004, and *I*^2^ = 0%). The LVEF was assessed in two studies (Kondo et al., 2019; Arenja et al., 2021), with 38 and 44 patients in the intervention and control groups, respectively (Fig. 5). The results revealed a nonsignificant difference between the intervention and control groups (MD = 1.94%, 95% CI [−3.91 to 7.79], *p* = 0.52, and *I*^2^ = 0%). To summarize, the intervention group exhibited significant improvements in 6MWD, leg muscle strength, and quality of life and nonsignificant improvement in LVEF. The *I*^2^ values for quality of life, leg muscle strength, and LVEF were low (*I*^2^ = 0%), indicating high consistency. Although the primary outcome (6MWD) showed high heterogeneity (*I*^2^ =71%), this value was within our pre-specified threshold, and a random-effects model was appropriately used to account for this variability.

**Table 1:** Characteristics of the included studies.

Study	Group	Sample size	Sex of participants (male/female)	Mean age (years)	NYHA Fc II/III/IV	LVEF (%)	Treatment	Outcome measure
De Araújo et al. (2012)	NMES	10	6/4	52.2	6/4/0	37.6	NMES + conventional rehabilitation	6MWD
	Control	10	6/4	49.5	6/4/0	38.2	Conventional rehabilitation	
Groehs et al. (2016)	NMES	15	14/1	54	0/0/15	22	NMES	6MWD, muscular strength and MLHFQ
	Control	15	13/2	49	0/0/15	22	Sham NMES	
Forestieri et al. (2018)	NMES	24	19/5	52.58	NA	27	NMES	6MWD
	Control	25	22/3	51.52	NA	25	Usual care	
Kondo et al. (2019)	NMES	34	25/9	75	0/17/17	37.5	NMES	LVEF
	Control	38	26/12	70	0/16/23	42.9	Sham NMES	
Arenja et al. (2021)	Low-intensity NMES	4	NA	76	NA	27.8	Low-intensity NMES	6MWD, LVEF and EQ-VAS
	High-intensity NMES	4	NA	78.8	NA	31.2	High-intensity NMES	
	Control	5	NA	83.9	NA	31.9	Usual care	
Poltavskaya et al. (2022)	NMES	22	15/7	64.5	0/17/5	32.3	NMES	6MWD and MLHFQ
	Control	23	11/12	68.9	0/19/4	30.8	Sham NMES	
Tanaka et al. (2022)	NMES	15	6/9	82.5	0/12/3	43.6	NMES + early rehabilitation	6MWD and QIS
	Control	16	8/8	83.3	0/14/2	43.2	Early rehabilitation	

**Notes.**

6MWD 6-min walking distance EQ-VAS EuroQol-visual analog scales LVEF left ventricular ejection fraction MLHFQ Minnesota Living with Heart Failure Questionnaire NMES neuromuscular electrical stimulation NYHA Fc New York Heart Association functional class QIS quadriceps isometric strength

**Table 2:** Characteristics of neuromuscular electrical stimulation in the included studies.

Study	Stimulus position	Intensity	Frequency	Pulse width	NMES duration
De Araújo et al. (2012)	Rectus femoris	Visible muscle contraction	20 Hz, biphasic	200 μs	2 ×60 min per day (morning and afternoon) until hospital discharge
Groehs et al. (2016)	Quadriceps + gastrocnemius	Visible muscle contraction	10 Hz	150 μs	60 min per day for 8–10 consecutive days
Forestieri et al. (2018)	Quadriceps + calf muscle	Visible muscle contraction	40 Hz, biphasic	400 μs	2 ×60 min per day until hospital discharge
Kondo et al. (2019)	Quadriceps femoris + triceps surae	10% of maximal voluntary contraction in the first and second pulse groups, 20% MVC in the third group and repeat throughout the session	First began with 200 Hz, then followed by 20 Hz	600 μs	30–60 min ×5 sessions per week during the entire hospitalization
Arenja et al. (2021)	Femoral and tibial muscles	Visible muscle contraction	25 Hz	1000 μs	30 min ×5 sessions per week × 6 weeks
			2.5 kHz (Russian stimulation)	10000 μs	16 min ×5 sessions per week ×6 weeks
Poltavskaya et al. (2022)	Femoral and tibial muscles	Visible muscle contraction	25 Hz	1000 μs	5 sessions per week until hospital discharge
Tanaka et al. (2022)	All leg muscle groups	Visible muscle contraction	20 Hz	250 μs	30–40 min per day, 5 days per week ×2 weeks

**Notes.**

NMES, neuromuscular electrical stimulation.

**Table 3:** Methodological quality assessment of included studies using the PEDro scale.

	1^*^	2	3	4	5	6	7	8	9	10	11	Total	Rating
De Araújo et al. (2012)	yes	yes	yes	yes	no	no	yes	yes	yes	yes	yes	8	Good
Groehs et al. (2016)	no	yes	no	yes	no	no	no	no	yes	yes	yes	5	Fair
Forestieri et al. (2018)	yes	yes	yes	yes	no	no	yes	no	no	yes	yes	6	Good
Kondo et al. (2019)	yes	yes	yes	yes	yes	no	yes	yes	no	yes	yes	8	Good
Arenja et al. (2021)	yes	yes	no	yes	no	no	yes	no	no	yes	yes	5	Fair
Poltavskaya et al. (2022)	yes	yes	yes	yes	yes	no	yes	yes	yes	yes	yes	9	Excellent
Tanaka et al. (2022)	yes	yes	yes	yes	no	no	no	no	yes	yes	yes	6	Good

**Notes.**

PEDro scale domains: 1, eligibility criteria and source of participants; 2, random allocation; 3, concealed allocation; 4, baseline comparability; 5, blinded participants; 6, blinded therapists; 7, blind assessors; 8, adequate follow-up; 9, intention-to-treat analysis; 10, between-group comparisons; 11, point estimates and variability.

* Not included in the calculation of the total score.

Three of the studies reported that both actual and sham neuromuscular electrical stimulation were well tolerated without any adverse events in all patients (De Araújo et al., 2012; Forestieri et al., 2018; Poltavskaya et al., 2022). Arenja et al. (2021) reported that one person could not tolerate the low-intensity neuromuscular electrical stimulation and withdrew from the study. Another study revealed that cerebral infarction developed in two intervention-group patients and one control-group patient (Kondo et al., 2019). In addition, in the control group, two patients developed lethal ventricular arrhythmia, and one died (Kondo et al., 2019). Heart rate and blood pressure variations were not significantly different between the groups, even with the use of vasodilators or inotropes (Kondo et al., 2019).

According to the GRADE criteria, the overall certainty of evidence ranged from low to moderate (Table 4). The evidence for quality of life was rated as moderate, downgraded primarily due to imprecision (small sample size). The evidence for 6MWD was rated as low, downgraded for both inconsistency (I^2^ = 71%) and imprecision. Similarly, the evidence for leg muscle strength and LVEF was rated as low, downgraded heavily for imprecision owing to the very small number of included trials and participants.

## Discussion

In this systematic review and meta-analysis, we compared the application of neuromuscular electrical stimulation to leg muscles in patients with acute heart failure with those undergoing control treatments. The results suggest that the use of neuromuscular electrical stimulation significantly improved the outcomes of 6MWD, quality of life, and leg muscle strength, but not LVEF. The observed improvements are not only statistically significant but also clinically meaningful. The pooled increase in 6MWD test was 69.92 m, which is more than double the 30–32 m change required to be considered clinically meaningful (Shoemaker et al., 2013). This was complemented by a large and significant effect on quality of life (SMD = 1.53), a key patient-reported outcome in heart failure management (Freedland, Rich & Carney, 2021). This indicates that the neuromuscular electrical stimulation intervention group experienced a significantly greater improvement in their health status compared to controls during hospitalization.

These clinical benefits are primarily driven by the effect of neuromuscular electrical stimulation on peripheral skeletal muscle. A key challenge in acute heart failure is the rapid onset of disuse atrophy and muscle wasting during hospitalization, a major complication linked to exercise intolerance and mortality (Lena, Anker & Springer, 2020; Saitoh et al., 2016). Neuromuscular electrical stimulation directly counteracts this by depolarizing motor nerves to trigger involuntary muscle contractions, activating otherwise inactive muscle fibers (Moritani, 2021; von Haehling et al., 2020). This process, which serves as a form of resistance training, explains the significant improvement in leg muscle strength (SMD = 0.77) observed in our analysis. This peripheral muscle adaptation—including enhanced skeletal muscle oxidative capacity, improved endothelial function, and a shift toward fatigue-resistant fiber types—reduces the metabolic burden on the heart during activity (Moritani, 2021; von Haehling et al., 2020). This ultimately translates into improved functional capacity, as evidenced by the clinically meaningful increase in 6MWD test.

**Table 4:** GRADE certainty of evidence summary.

**Outcomes**	**Number of participants (studies)**	**Effect Estimate (95% CI)**	**Certainty of the evidence (GRADE)**	**Reason for downgrading**
6-min walking distance	184 (6 RCTs)	MD = 69.92 (95% CI [32.17–107.68])	⨁⨁○○ Low	Inconsistency (−1)^*^ Imprecision (−1)^**^
Quality of life	84 (3 RCTs)	SMD = 1.53 (95% CI [1.03–2.03])	⨁⨁⨁○ Moderate	Imprecision (−1)^**^
Leg muscle strength	61 (2 RCTs)	SMD = 0.77 (95% CI [0.25–1.29])	⨁⨁○○ Low	Imprecision (−2)^***^
Left ventricular ejection fraction	82 (2 RCTs)	MD = 1.94 (95% CI [−3.91 to 7.79])	⨁⨁○○ Low	Imprecision (−2)^***^

**Notes.**

* Downgraded by 1 level due to substantial heterogeneity (I^2^ = 71%).

** Downgraded by 1 level due to small sample size limiting the precision of the estimate.

*** Downgraded by 2 levels due to very small sample size and few included trials.

Abbreviations: CI, confidence interval; MD, mean difference; SMD, standardized mean difference.

The finding that neuromuscular electrical stimulation did not significantly improve LVEF is also consistent with current physiological understanding. Neuromuscular electrical stimulation is a peripheral intervention that primarily targets skeletal muscle. Its benefits are thought to arise from peripheral adaptations—such as improved endothelial function and muscle oxidative capacity—which reduce the afterload on the heart, rather than direct inotropic effects on the myocardium. Mechanistically, central cardiac reverse remodeling, which manifests as an improved LVEF, typically requires long-term hemodynamic offloading and sustained neurohormonal modulation. The short intervention duration during an acute hospital stay (typically 1 to 2 weeks in the included trials) is simply insufficient to induce such macroscopic structural changes in the heart. Furthermore, there are inherent limitations in measuring central cardiac function changes in this specific acute setting. LVEF is a highly load-dependent parameter, meaning its measurement can be significantly confounded by the rapid shifts in volume status and intense diuretic therapy that characterize acute heart failure management. Thus, improvements in functional capacity (6MWD) are likely decoupled from changes in central cardiac function (LVEF) in this context.

Regarding the intervention’s feasibility, neuromuscular electrical stimulation appears both safe and efficient in the acute heart failure setting. No serious adverse events were directly attributable to the intervention. While some events like cerebrovascular incidents were reported, they occurred in both intervention and control groups, with more serious events (*e.g.*, lethal arrhythmia, death) occurring only in the control group, suggesting these were unrelated to neuromuscular electrical stimulation. Furthermore, significant functional improvements were seen after relatively short intervention periods, typically around 2 weeks. This contrasts sharply with the 5–10-week programs common in chronic heart failure studies, implying that acute heart failure patients may achieve rapid benefits, though further research is needed to confirm this.

The findings of this review are crucial as they establish the utility of neuromuscular electrical stimulation in a population often excluded from rehabilitation (Warraich et al., 2018; Reeves et al., 2016). While exercise-based cardiac rehabilitation is a Class IA recommendation and neuromuscular electrical stimulation is known to be effective in stable chronic heart failure (Kamiya et al., 2020; Yancy et al., 2017), these interventions are often unfeasible for acute heart failure patients (Grace, Kotseva & Whooley, 2021; Pack et al., 2019; Supervia et al., 2019). Many in the acute phase are unable or unwilling to participate in conventional exercise due to severe symptoms and functional deterioration, leading to extremely low inpatient cardiac rehabilitation utilization (Grace, Kotseva & Whooley, 2021; Pack et al., 2019; Supervia et al., 2019). Furthermore, most trials in stable chronic heart failure explicitly excluded acute heart failure patients, leaving a gap in evidence (Wang et al., 2022; Gomes Neto et al., 2016; Sbruzzi et al., 2010). Our results position neuromuscular electrical stimulation as a viable alternative or adjunct therapy for this vulnerable population. It can serve as a “bridge therapy” during early hospitalization, improving physical function and muscle strength to a level where patients may eventually be able and willing to transition to conventional exercise-based cardiac rehabilitation.

This meta-analysis also possesses several strengths. It is, to our knowledge, the first systematic review to focus exclusively on the effects of neuromuscular electrical stimulation in the acute heart failure population, a critically ill group often excluded from rehabilitation studies. By adhering to PRISMA and Cochrane guidelines and conducting a comprehensive search of multiple databases, this review provides the most current synthesis of evidence for this specific clinical application.

This study has some limitations. First, while the search was comprehensive, the primary limitation is the small cumulative sample size (*N* = 256 total participants) across the seven included trials. This small sample size limits the statistical power of the analysis and the precision of the pooled estimates. Second, different protocols of neuromuscular electrical stimulation, including frequencies, durations, numbers of training sessions, and different stimulated muscles, were used, leading to high heterogeneity. However, owing to the limited number of included trials (maximum *n* = 6 for any single outcome), we were unable to conduct meaningful subgroup analyses to statistically explore these sources of heterogeneity without a high risk of false-positive or false-negative findings. Third, we were unable to formally assess the risk of publication bias using a funnel plot because the number of included trials (*n* = 7) was below the recommended threshold of 10, and this potential bias should be considered when interpreting our findings. Fourth, adequate blinding of participants and personnel is inherently difficult in neuromuscular electrical stimulation trials, and the lack of blinding in several included studies may introduce performance and detection biases. Fifth, there was a prominent sex bias in the included trials, with the majority of enrolled participants being male (Table 1), which may limit the generalizability of these findings to female patients with acute heart failure. Finally, the analyses for secondary outcomes of leg muscle strength and LVEF were based on data from only two trials each, making these findings preliminary and warranting cautious interpretation. Additional high-quality large-scale RCTs with long-term follow-up periods and well-defined protocols of neuromuscular electrical stimulation are required to overcome these limitations.

## Conclusion

The results of this meta-analysis indicate that neuromuscular electrical stimulation is a safe and promising intervention for improving functional capacity and quality of life in patients with acute heart failure. Preliminary evidence from a limited number of trials also suggests a potential benefit for leg muscle strength. It can be considered an alternative or adjunct therapy for those who cannot tolerate the intensity of conventional exercise-based rehabilitation therapies. However, given the limited scale and potential risk of bias in this study, further high-quality RCTs with larger sample sizes are needed for verification.

##  Supplemental Information

10.7717/peerj.21352/supp-1 Supplemental Information 1 PRISMA checklist

10.7717/peerj.21352/supp-2 Supplemental Information 2 Systematic review and/or meta-analysis rationale

10.7717/peerj.21352/supp-3 Supplemental Information 3 Data

## Additional Information and Declarations

### Data Availability

The following information was supplied regarding data availability: The data is available in the Supplementary File.
