# Supplementary material for: A systematic review and meta-analysis of randomized controlled trials on the effects of neuromuscular electrical stimulation in patients with acute heart failure
Source: PeerJ. 2026 Jun 9;14:e21352. doi: 10.7717/peerj.21352 (PMC13262542; doi:10.7717/peerj.21352)
Supplement: Supplemental Information 2 [file peerj-14-21352-s002.docx]

**1. Rationale for conducting the systematic review / meta-analysis**

Acute heart failure leads to severe functional decline, immobilization, and poor quality of life, yet the feasibility of conventional exercise-based rehabilitation in this population is limited. Neuromuscular electrical stimulation (NMES) offers a potential alternative by safely inducing muscle contractions without increasing cardiopulmonary burden. Although several trials have investigated NMES in acute heart failure, their findings remain inconsistent and have not been systematically synthesized. Therefore, a systematic review and meta-analysis was needed to clarify the overall effectiveness of NMES in this specific acute inpatient population.

**2. Contribution to existing knowledge**

This review is the first to focus exclusively on NMES in patients hospitalized with acute heart failure—a population distinct from the stable chronic heart failure cohorts included in previous systematic reviews. By integrating the most up-to-date randomized controlled trials, our study provides the most comprehensive pooled estimates of NMES effects on functional capacity, quality of life, and muscle strength during acute hospitalization. The findings fill an important evidence gap, highlight NMES as a feasible alternative for patients unable to perform conventional rehabilitation, and identify areas requiring further high-quality research.
